# Vitamin D Deficiency and Associated Risk Factors in Muslim Housewives of Quetta, Pakistan: A Cross-Sectional Study

**DOI:** 10.7759/cureus.17643

**Published:** 2021-09-01

**Authors:** Taimoor Hussain, Abdul Habib Eimal Latif, Sheza Malik, Sami Raza, Tooba Saeed, Asjad Salman Zahid, Kefayatullah Nazary, Mohammad mohsin Arshad, Rajeswari Khan, Khalida Walizada, Ahsan Wahab

**Affiliations:** 1 Neurology/General Medicine, Bolan Medical College, Quetta, PAK; 2 Internal Medicine, Kabul University of Medical Sciences, Kabul, AFG; 3 Medicine, Army Medical College Rawalpindi, Rawalpindi, PAK; 4 Orthopedics, Shaheed Mohtarma Benazir Bhutto Government Hospital Quetta, Quetta, PAK; 5 Medicine and Surgery, Kabul University of Medical Sciences, Kabul, AFG; 6 Internal Medicine, Allama Iqbal Memorial Teaching Hospital, Lahore, PAK; 7 Internal Medicine, Kabul University of Medical Science, Kabul, AFG; 8 Medicine and Surgery, Multan Medical and Dental College, Multan, PAK; 9 Medicine and Surgery, Hospital College of Medicine and Sagore Dutta Hospital, Kolkata, IND; 10 Neurological Surgery, Ali Abad Teaching Hospital Karte Sakhi Kabul Afghanistan, Kabul, AFG; 11 Internal Medicine Department, Baptist Medical Center South, Montgomery, USA

**Keywords:** vitamin d deficiency, vitamin d, vitamin d deficiency in housewives, vitamin d deficiency in muslim housewives, vitamin d status in pakistan

## Abstract

Background

Vitamin D (Vit-D) plays a central role in calcium homeostasis and maintains skeletal integrity. Housewives in Quetta, Pakistan are at increased risk of vitamin D deficiency (VDD). They spend a greater part of their day in cleaning, washing, cooking, managing daily groceries, and other household chores. Thus, little time is left for self-care and outdoor activities. They wear hijab and have very little exposure to sunlight. In addition, their diet is deficient in Vit-D-rich food items, rendering them at high risk of VDD. Fear of getting tanned, melasma, and preference for a fair complexion further limit their sun exposure. This study evaluates the prevalence of VDD in housewives and determines its various risk factors to recommend screening guidelines for VDD.

Methods

A cross-sectional study was performed between November 2020 and April 2021 and recruited housewives aged >18 from the outpatient department of a tertiary care hospital in Quetta. Informed consent was obtained from all participants. VDD was defined as a serum 25(OH)-D level <20 ng/mL (50 nmol/L). Sociodemographic variables and information about the dietary habits, perception, attitudes towards sunlight, and daily duration of sunlight exposure were collected. Mean and standard deviation (SD) were calculated for continuous variables and counts, and proportions were calculated for categorical variables like education, age. Univariate and multivariate logistic regression analyses were performed to determine the risk factors and associations of VDD. Data were analyzed by SAS/STAT software (version 9.4).

Results

Among 151 housewives, 58.9% of housewives had VDD. VDD group had a higher proportion of females aged 18-30 years and a lower proportion of graduates. The reported use of Vit-D supplements was much lower in the VDD group compared with the non-deficient group, 38.2% versus 71.0 %, P-value <0.001. History of fragility fractures was reported by 10.1% of housewives in the VDD group compared to 4.8% in the non-deficient group, P-value: 0.03. Around 77.5% of housewives in the VDD group spent 15 minutes or less outdoors versus 51.6% in the non-deficient group; 55.1% of housewives in the VDD group reported that they never consumed milk versus 17.7 % in the non-deficient group, P-value <0.00001. In the univariate logistic regression model, housewives with an 11-12th grade of education had 4.80-fold higher odds of VDD compared to those who had undergraduate or graduate degrees (OR: 4.80, 95 % CI: 1.07-21.45). Housewives who never consumed milk had 9.72-fold (95 % CI: 3.69-25.58) higher odds of VDD compared to those who consumed milk on daily basis. Odds of VDD were 3.61-fold (95% CI: 1.06-12.31) higher in those who never consumed fish as compared to those who ate fish at least 1-2 days/week. In multivariate logistic regression, age group 18-30 (OR: 17.07, 95% CI: 1.18-246.86), and never consuming milk (OR: 7.33, 95 % CI: 1.99-26.89) were independently associated with VDD.

Conclusion

VDD is highly prevalent (58.9%) in housewives of Quetta. It is the need of time to increase awareness regarding the health benefits, sources, and deficiency symptoms of Vit-D. Our study revealed VDD in housewives irrespective of education and income. Dietary supplementations were greater predictors of VDD. Daily sun exposure should be encouraged, and food items should be fortified with Vit-D. Recommendations for Vitamin D screening would be a good step, especially in Muslim housewives.

## Introduction

Vitamin D (Vit-D) has a pivotal role in the maintenance of skeletal integrity. Synthesis of its inert form begins after skin exposure to ultraviolet-B (UVB) radiations. It is then hydroxylated in the liver and kidney to form its active product, 1, 25-dihydroxy vitamin D. Synthesis of Vit-D is regulated by calcium and phosphorus homeostatic mechanisms. Vit-D deficiency (VDD) is a function of several factors, such as race/ethnicity, age, dietary habits, body mass index (BMI), sunscreen use, UVB exposure, and clothing patterns [1]. VDD is associated with numerous negative health consequences such as osteomalacia, osteoporosis, muscle weakness, and falls [2-5]. Vit-D protects against the development of many cancers such as colon and breast cancer [6]. VDD has associations with both obesity and diabetes [6]. Fat content in a body is inversely correlated with Vit-D levels, and VDD may contribute to the development of both types I and II diabetes [7]. Through its role in calcium homeostasis indirectly and receptors on B-islet cells of the pancreas directly, vitamin D is involved in insulin secretion [8-10]. Improvement in glycemic control and reduction in risk of type 1 diabetes has been observed by supplementation of vitamin D and calcium in meta-analysis [9,11]. Vit-D boosts the immune responses and a few studies have reported an enhanced risk of acquiring tuberculosis and other respiratory infections because of VDD [12-16]. Hypovitaminosis D has a statistically significant inverse relationship with hypertension, obesity, diabetes, and hypertriglyceridemia [17-19]. All these disorders are associated with increased cardiovascular risk and related mortality [17-19].

In our study, we evaluated the 25(OH)-D concentrations, lifestyle habits, perception, attitude and exposure to the sun, dietary habit, and also several demographic variables from a sample of housewives in Quetta, the capital of Baluchistan province of Pakistan, to examine the prevalence of VDD and its risk factors. The latitude and the longitude of Quetta, Pakistan are 30.183270, and 66.996452, respectively. The average sunshine in Quetta from October to February is 7.1-9.9 hours, respectively [20].

Housewives are at increased risk of VDD in Muslim society due to sociocultural factors. Housewives in Quetta, spend a greater part of their day in cleaning, washing, cooking, managing daily groceries, utilities, and other household chores. Once they are done with household chores, they attend communal festivities, meet and greet, and other obligatory gatherings. Thus, little time is left for outdoor activities. They are usually clothed from head to toe, draped in “chador” or “hijab” per local customs and religion. They do not have time or the opportunity to soak in the sun and even if they do, very little of the sun's rays make it to their skin. Their diet is deficient in Vit-D-rich foods which further aggravates VDD. Fear of getting tanned or dark complexion, skin blemishes, or melasma from sun exposure further limits their sun exposure. A great majority of the population is unaware of Vit-D sources, benefits, and deficiency symptoms. Families live in small single or double-story buildings and the city is densely populated. The houses have little to no space for sun exposure and even if they do women do not properly expose their skin to the sun due to hijab. Thus, they are at increased risk of VDD. This study highlights a high prevalence of VDD in housewives as a public health concern and evaluates its various risk factors to recommend screening guidelines.

## Materials and methods

A cross-sectional study was performed between November 2020 and April 2021. Convenience sampling was used. Inclusion criteria were housewives of all ethnicities who gave consent and aged >18 years, visiting the outpatient department of a tertiary care hospital in Quetta, Pakistan. Pregnant or lactating females, patients previously treated for rickets and osteomalacia, those on certain drugs such as anti-epileptics/steroids/hormone replacement/bisphosphonates/oral contraceptives, and those with a history of fractures in the last one year were not recruited. IRB approval was obtained from the local review committee of the hospital. Informed consent was obtained from all participants.

Serum 25-hydroxyvitamin D [25(OH)-D] level was measured using COBAS C-6000 by the electrochemiluminescence immunoassay (ECLI) method. VDD was defined as a serum 25(OH)-D level <20 ng/mL (50 nmol/L) as per Endocrine Society Clinical Practice Guidelines [21]; those with 25(OH)-D level above 20 ng/mL were categorized as non-deficient.

Sociodemographic variables (age, sex, education, household income, and occupation) and information about the dietary habits, perception, attitudes towards sunlight, and daily duration of sunlight exposure were collected. Height and weight were recorded to calculate BMI. History of body aches was also taken. Mean and standard deviation (SD) were calculated for continuous variables and counts, and proportions were calculated for categorical variables. Chi-square and t-test were performed to check the distribution of categorical and continuous variables, respectively, across Vit-D deficient and non-deficient individuals. Two-tailed p-value >0.05 was considered statistically significant for a different distribution of these variables between the two groups. Univariate and multivariate logistic regression analyses were performed to determine the risk factors of VDD. Data were analyzed by SAS/STAT software (version 9.4), Copyright © 2016, SAS Institute Inc., Cary, NC, USA.

## Results

Among 151 housewives, the prevalence of VDD was 58.9%. About 90% of housewives were of Hazara ethnicity. Fifty-three housewives (35.1%) were aged above 50 years. Most of the housewives belonged to a low income (34.4%) and uneducated (43.0%) category. Mean Vit-D ± SD was 11.5 ng/ml ± 4.5 and 35.9 ng/ml ± 16.2 in Vit-D deficient and non-deficient groups, respectively (P-value <0.0001). Mean BMI ± SD was 28.4 ± 6.2 and 26.7 ± 5.4 in the Vit-D deficient and non-deficient group, respectively (P-value: 0.0983).

Demographics characteristics of vitamin D deficient and non-deficient group

Eighty-nine (58.9%) housewives had VDD whereas 62 (41.1%) housewives were non-deficient of Vit-D. VDD group had a higher proportion of females aged 18-30 years, 16.9% compared to 4.8% in the non-deficient group (P-value: 0.1083). Ethnicity and monthly income were not significantly different between the two groups. Forty percent of females in the VDD group had a monthly income of less than 30,000 PKR (Pakistani rupees) compared to 30% of females without VDD. VDD group had a lower proportion of graduates and those with an undergraduate degree, i.e., 6.3% compared to 17.2% in non-deficient, P-value: 0.0765. Housewives in the VDD group were more obese but results did not reach significance. The reported use of Vit-D supplements was much lower in the VDD group compared with the non-deficient group, 38.2% versus 71.0 %, P-value <0.001. The proportion of Vit-D injection was also much lower in the VDD group (30.3%) versus non-deficient (64.5%), P-value <0.001. History of fragility fractures was reported by 10.1% of housewives in the VDD group compared to 4.8% in the non-deficient group, P-value: 0.03. History of C-sections, pregnancies, menopause, and body aches were not different between groups. Around 77.5% of housewives in the VDD group spent 15 minutes or less outdoors versus 51.6% in the non-deficient group. Sun exposure avoidance behavior was more prevalent in Vit-D deficient housewives, 51.7 % versus 43.6 % in non-deficient housewives, although the result did not reach significance. History of menopause and pregnancy were not associated with VDD (p-value: 0.4379 and 0.8859, respectively); 55.1% of housewives in the VDD group reported that they never consumed milk versus 17.7% in the non-deficient group, P-value <0.00001. Fish consumption was also 10% lower in housewives with VDD compared to those without VDD, 4.5% vs 14.5%, P-value 0.0308. Detailed demographic, behavioral, and dietary attributes of the sample stratified by Vit-D deficiency are shown in Table 1.

**Table 1:** Sociodemographic, behavioral, and dietary attributes of vitamin D deficiency among housewives of Quetta. *Missing values: Education: 13, monthly income: 6, history of C-sections: 14, usual practice in sunlight: 1, history of body aches: 4. Percentages are given in parenthesis.

Variables	Total sample n=151 (%)	Vitamin D deficient n=89 (%)	Vitamin D non-deficient n=62 (%)	P-value
Age group				0.1083
18–30 years	18 (11.9)	15 (16.9)	3 (4.8)	
31–40 years	44 (29.1)	22 (24.7)	22 (35.5)	
41–50 years	36 (23.8)	22 (24.7)	14 (22.6)	
>51 years	53 (35.1)	30 (33.7)	23 (37.1)	
Ethnicity				0.4753
Hazara	137 (90.7)	82 (92.1)	55 (88.7)	
Non-Hazara	14 (9.3)	7 (7.9)	7 (11.3)	
Education*				0.0765
Uneducated	65 (47.1)	34 (42.5)	31 (53.5)	
Up to 5^th^ grade	10 (7.3)	7 (8.8)	3 (5.2)	
6^th^ grade to 10^th^ grade	31 (22.5)	22 (27.5)	9 (15.5)	
11^th^ to 12^th^ grade	17 (12.3)	12 (15.0)	5 (8.6)	
Undergraduate or graduate degree	15 (10.9)	5 (6.3)	10 (17.2)	
Monthly income*				0.2464
<30,000 PKR	52 (35.9)	34 (40.0)	18 (30.0)	
31,000–50,000 PKR	48 (33.1)	29 (34.1)	19 (31.7)	
>50,000 PKR	45 (31.0)	22 (25.9)	23 (38.3)	
Vitamin D level mean (ng/ml) ± SD	21.5±16.2	11.5±4.5	35.9±16.2	<0.0001
BMI mean (kg/m^2^) ± SD	27.7±6.0	28.4±6.2	26.7±5.5	0.0983
Obesity				0.1644
Non-obese (BMI <24.9 kg/m^2^)	58 (38.4)	29 (32.6)	29 (46.8)	
Overweight (BMI 25–29.9 kg/m^2^)	47 (31.1)	32 (35.9)	15 (24.2)	
Obese (BMI>30 kg/m^2^)	46 (30.5)	28 (31.5)	18 (29.0)	
History of pregnancy				0.8859
Yes	137 (90.7)	81 (91.0)	56 (90.3)	
No	14 (9.3)	8 (9.0)	6 (9.7)	
History of C-sections*				0.3043
Yes	17 (12.4)	12 (14.8)	5 (8.9)	
No	120 (87.6)	69 (85.2)	51 (91.1)	
History of menopause				0.4379
Yes	53 (35.1)	29 (32.6)	24 (38.7)	
No	98 (64.9)	60 (67.4)	38 (61.3)	
History of vitamin D supplements intake				<0.0001
Yes	78 (51.7)	34 (38.2)	44 (71.0)	
No	73 (48.3)	55 (61.8)	18 (29.0)	
Vitamin D injections				<0.0001
Yes	67 (44.4)	27 (30.3)	40 (64.5)	
No	84 (55.6)	62 (69.7)	22 (35.5)	
History of fractures				0.0309
No fractures	132 (87.4)	75 (84.3)	57 (91.9)	
High impact fractures	7 (4.6)	5 (5.6)	2 (3.2)	
Fragility or pathological fractures	12 (8.0)	9 (10.1)	3 (4.8)	
History of body aches*				0.3654
Yes	135 (91.8)	80 (89.9)	55 (94.8)	
No	12 (8.2)	9 (10.1)	3 (5.2)	
Areas of skin exposed to sunlight face and hands exposed	151 (100)	89 (100)	62 (100)	NA
Time spent outdoors on weekdays				0.0038
1-hour or more	9 (5.9)	4 (4.5)	5 (8.1)	
16 minutes to less than 1 hour	41 (27.2)	16 (18.0)	25 (40.3)	
15 minute or less	101 (66.9)	69 (77.5)	32 (51.6)	
Usual practice in sunlight*				0.4095
Do not go outside	22 (14.7)	15 (17.1)	7 (11.3)	
Cover up/wear clothing	56 (37.3)	30 (34.1)	26 (41.9)	
Seek direct sunlight	36 (24.0)	19 (21.6)	17 (27.4)	
Shade	36 (24.0)	24 (27.3)	12 (19.4)	
Sun exposure avoidance behavior				0.3250
Yes	73 (48.3)	46 (51.7)	27 (43.6)	
No	78 (51.7)	43 (48.3)	35 (56.4)	
Egg’s intake behavior				0.1259
1–2 days per week	64 (42.4)	39 (43.8)	25 (40.3)	
3–4 days per week	19 (12.6)	8 (9.0)	11 (17.7)	
5–6 days per week	8 (5.3)	3 (3.4)	5 (8.1)	
Daily	13 (8.6)	6 (6.7)	7 (11.3)	
Never	47 (31.1)	33 (37.1)	14 (22.6)	
Milk intake behavior				<0.0001
1–2 days per week	36 (23.8)	22 (24.7)	14 (22.6)	
3–4 days per week	14 (9.3)	6 (6.7)	8 (12.9)	
5–6 days per week	6 (4.0)	1 (1.1)	5 (8.1)	
Daily	35 (23.2)	11 (12.4)	24 (38.7)	
Never	60 (39.7)	49 (55.1)	11 (17.7)	
Fish intake behavior				0.0308
1–2 days per week	13 (8.6)	4 (4.5)	9 (14.5)	
Never	138 (91.4)	85 (95.5)	53 (85.5)	

Demographic, dietary, and behavioral determinants of vitamin D deficiency

In the univariate logistic regression model (Table 2), housewives with an 11-12th grade of education had 4.80-fold higher odds of VDD compared to those with undergraduate or graduate degrees (OR: 4.80, 95 % CI: 1.07-21.45). Those who did not report consuming multivitamins, vitamin D supplements, or vitamin D injections had 2.21-fold (95 % CI: 1.10-4.44), 4.18-fold (95 % CI: 2.10-8.32), and 3.95-fold (95 % CI: 1.97-7.93) higher odds of VDD compared to those who reported using those. Housewives who never consumed milk had 9.72-fold (95 % CI: 3.69-25.58) higher odds of VDD compared to those who consumed milk on daily basis. Odds of VDD were 3.61-fold (95% CI: 1.06-12.31) higher in those who never consumed fish as compared to those who ate fish at least one to two days/week. Those who reported less than 15 min daily sun exposure had 2.70-fold (CI: 0.68-10.71) higher odds of VDD as compared to those who had one hour or more daily sun exposure, however, the result did not reach a significance level. Similarly, housewives who did not go out or were covered up/wore complete clothing had a higher risk of VDD, but results did not reach significance. The presence of body ache could not predict VDD (OR: 0.49, 95% CI: 0.13-1.87).

**Table 2:** Univariate logistic regression model for determinants of vitamin D deficiency.

Covariates	Subdivisions	Odds ratio of vitamin D deficiency (95% Confidence interval)
Age groups	18–30 years	3.83 (0.99–14.84)
	31–40 years	0.77 (0.34–1.71)
	41–50 years	1.21 (0.51–2.85)
	>50 years	REF
Monthly income	<30,000 PKR	1.98 (0.87–4.47)
	31,000-50,000 PKR	1.60 (0.70–3.63)
	>50,000 PKR	REF
Education	Uneducated	2.19 (0.68–7.13)
	Up to 5^th^ grade	4.67 (0.83–26.24)
	6^th^ grade to 10^th^ grade	4.89 (1.30–18.38)
	11^th^ to 12^th^ grade	4.80 (1.07–21.45)
	Undergraduate or graduate degree	REF
Obesity	Obese	1.56 (0.71–3.41)
	Overweight	2.13 (0.96–4.75)
	Non-obese	REF
Multivitamin intake	No	2.21 (1.10–4.44)
	Yes	REF
Vitamin D injections	No	4.18 (2.10–8.32)
	Yes	REF
Vitamin D supplements	No	3.95 (1.97–7.93)
	Yes	REF
Time spent outdoors on workdays	15 minutes or less	2.70 (0.68–10.71)
	16 minutes or less than 1 hour	0.80 (0.19–3.43)
	1 hour or more	REF
Sunlight avoidance exposure behavior	Yes	1.39 (0.72–2.66)
	No	REF
Usual practice in sunlight	Do not go outside	1.92 (0.63–5.82)
	Cover up and wear clothing	1.03 (0.45–2.39)
	Use shade while in the sun	1.79 (0.69–4.641)
	Seek direct sunlight	REF
History of body aches	Yes	0.49 (0.13–1.87)
	No	REF
Eggs intake	1–2 days per week	1.82 (0.55–6.05)
	3–4 days per week	0.85 (0.21–3.51)
	5–6 days per week	0.70 (0.12–4.23)
	Never	2.75 (0.78–9.66)
	Daily	REF
Milk intake	1–2 days per week	3.43 (1.29–9.13)
	3–4 days per week	1.64 (0.46–5.87)
	5–6 days per week	0.44 (0.05–4.19)
	Never	9.72 (3.69–25.58)
	Daily	REF
Fish intake	Never	3.61 (1.06–12.31)
	1–2 days per week	REF

A multivariate logistic regression model to evaluate independent determinants of VDD is given in Table 3. Those determinants with significant association with VDD in the univariate model plus monthly income were added in the multivariate model. Age group 18-30 years (OR: 17.07, 95% CI: 1.18-246.86), and never consuming milk (OR: 7.33, 95 % CI: 1.99-26.89) were independently associated with VDD.

**Table 3:** Multivariate logistic regression model for determinants of vitamin D deficiency.

Covariate	Subdivisions	Odds ratio for vitamin D deficiency (95% confidence interval)
Age groups	18–30 years	17.07 (1.18–246.86)
	31–40 years	0.61 (0.18–2.11)
	41–50 years	0.82 (0.23–2.90)
	>50 years	REF
Monthly income	<30,000 PKR	0.55 (0.15–1.98)
	31,000–50,000 PKR	0.973 (0.31–3.05)
	>50,000 PKR	REF
Education	Uneducated	1.76 (0.35–8.87)
	Up to 5th grade	2.77 (0.28–26.94)
	6th grade to 10th grade	1.49 (0.26–8.48)
	11th to 12th grade	1.89 (0.23–15.37)
	Undergraduate or graduate degree	REF
Multivitamin intake	No	0.76 (0.23–2.47)
	Yes	REF
Vitamin D injections	No	2.99 (0.74–12.07)
	Yes	REF
Vitamin D supplements	No	3.16 (0.66–15.22)
	Yes	REF
Milk intake	1–2 days per week	2.36 (0.65–8.56)
	3–4 days per week	0.66 (0.13–3.28)
	5–6 days per week	0.17 (0.01–2.20)
	Never	7.33 (1.99–26.89)
	Daily	REF
Fish intake	Never	5.43 (0.97–30.52)
	1–2 days per week	REF

## Discussion

Pakistan is a country that has adequate sunlight exposure making skin production of vitamin D quite possible throughout the year. Despite this, the prevalence of VDD among females is remarkably high in Pakistan (66.8% for non-pregnant females versus 68.9% for pregnant) according to the National Nutritional Survey of Pakistan 2011 [22]. Based on the survey, VDD is widespread across all the provinces in Pakistan (54.6-80.9%) including Kashmir and Gilgit Baltistan, and the situation is worse in urban areas (72.5% in urban versus 64.3% in rural). According to a report of the Food and Agricultural Organization of the United Nations, about 40 million people in Pakistan are food insecure [23]. In the National Nutritional Survey of Pakistan 2018, 36.9% of the population was estimated to be food insecure and 20.9% of the population was malnourished [24]. Food insecurity in the country is mainly due to poverty, and disproportionately affects vulnerable groups such as women [24]. Poverty is directly related to inadequate food consumption and/or consumption of food with poor nutritional value. Researchers from Pakistan have previously investigated the prevalence of VDD in different regions of Pakistan. In a Karachi-based study of 577 patients (72.7% females, 49.9% housewives) conducted by Raza et al., 51.6% of patients had VDD (44.2% in Summer and 60.3% in Winter). VDD was most prevalent among housewives when compared with other professions, 59.4% in housewives followed by 56.4% for students and 40.5% in unemployed [25]. In another study from Karachi, VDD was reported to be present in 84% of the asymptomatic adult population (62.3% females) [26] and 91% among community-dwelling premenopausal females [27]. A study conducted in Lahore by Junaid et al. reported a VDD prevalence of 73% among women of childbearing age [28]. In an NHANES-based study for 2011-2012, only 39.9% of the interviewed US population had VDD [29], much lower than that estimated by Raza et al. and Junaid et al. [25,28].

Our study also reports a very high prevalence of VDD (58.9%) among screened housewives supported by the previous literature. Studies conducted elsewhere in Asia such as from Bangladesh and India have reported similarly a high prevalence of VDD [30,31]. Nationwide studies in India have previously reported VDD prevalence as high as 70-100% of the population. VDD was reported to be highly prevalent across India particularly in pregnant and lactating females [32]. A study on Kuwaiti women reported higher levels of markers of bone turnover among those women who wore hijab or veils versus those who did not. The prevalence of VDD was also much higher in these women compared to those who wore western-style clothes [33]. Clothing styles have been reported to be associated with VDD in Muslim countries with adequate sun exposure such as Turkey and Jordan [34,35]. Clothing style is also associated with VDD among Arab/Muslim females who live in Western countries [36,37]. In our study, 100% of housewives reported that only hands and faces are exposed in their usual clothing style. Our study revealed that VDD is prevalent irrespective of education or income but housewives with lower education might be at higher risk of VDD.

Artificial supplementation of Vit-D in the form of multivitamins, oral, and injectable vitamin D is associated with a lower risk of VDD. Lack of milk and fish in the diet is associated with a higher risk of VDD. A study by Junaid et al. found an association between VDD and illiteracy but found no association between VDD and monthly income. Similar to our study, lack of multivitamin intake was a negative predictor of VDD [27].

In multivariate analysis, we found that females aged 18-30 and no milk use were independent predictors of VDD. Previously, a number of studies have reported an association between age and VDD [38-42]. A study in children and adults from Saudi Arabia found a modest but positive correlation between Vit-D level and dairy product consumptions [43]. The cause of this modest relationship was thought to be an inconsistent fortification of dairy products with Vit-D [44].

There are multiple diverse determinants of VDD such as age, area of residence (urban vs suburban or rural), and housing structures. Females who lived in small houses of Karachi in a study were more likely to have VDD compared to those living in affluent houses [40]. Body ache was not a predictor of Vit-D in our study, a finding similar to that reported by Reza et al. [24], and contrary to what was reported by Junaid et al. [26]. Another probable explanation of prevalent VDD is increased use of technology such as television, computers, and mobile devices making them inclined to stay indoors as compared to earlier generations and, consequently, decreasing their sunlight exposure. VDD is also influenced by variation in the genes encoding the vitamin D 25-hydroxylase enzyme CYP2R1, the vitamin D binding protein (DBP), and the vitamin D receptor (VDR), which was not determined in our study [45,46]. Quetta is one of the highly polluted cities in the world [47]. The Vit-D synthesis also depends on UVB light exposure, which in turn is reduced by the pollutants in the atmosphere [48]. Air pollution can be a main factor of VDD in polluted cities [49]. Further studies are needed with a larger sample size, including seasonal variations, genetic determinants, a more detailed systematic record of sun exposure duration, consumption of dietary sources of Vit-D, multivitamins, and Vit-D supplements use.

The reason for selecting 30 ng/mL as the cutoff value for defining optimal Vit-D status is based on multiple cross-sectional examinations of the relationship between serum PTH and 25(OH) D levels exhibiting a plateau in PTH suppression when the 25(OH) D level reaches approximately 30 ng/mL [50]. However, the proposed definition of VDD as a serum Vit-D <30 ng/ml (75 nmol/liter), based on serum PTH suppression, is not supported by the literature review [51]. Therefore, the seemingly prevalent VDD pandemic would be less prevalent if different serum vitamin D level was used to define sufficiency, insufficiency, or deficiency.

Nevertheless, VDD is a prevalent health issue in the Pakistani population as compared to others. A Norwegian study has observed a high prevalence of VDD in immigrants of Pakistani origin as compared to the Norwegians and other immigrant groups including immigrants living in Oslo from Turkey, Srilanka, Iran, and Vietnam [52,53]. This fact is in contradiction with the assumption that because Pakistan has adequate sunlight exposure it is unlikely that Pakistan would have VDD as a prevalent health problem.

This study has a number of limitations. First, we did not collect any information on participants’ individual skin pigmentation on any skin pigmentation scale. Second, information collected depends upon the participants’ recall or memory and is prone to recall bias. Third, our sample size was small and could have been increased. Fourth, the study population involves only one government hospital and the population visiting this hospital could be different from those visiting private clinics or other hospitals.

## Conclusions

VDD is highly prevalent (58.9%) in housewives of Quetta Pakistan. It is the need of time to increase awareness regarding the health benefits, sources, and deficiency symptoms of Vit-D. Our study revealed Vit-D deficiency in housewives irrespective of education and monthly income. Daily sun exposure should be encouraged, and food items should be fortified with Vit-D. Recommendations for Vit-D screening would be a good step, especially in Muslim housewives who do not consume milk or fish.
